# GABA in the anterior cingulate cortex mediates the association of white matter hyperintensities with executive function: a magnetic resonance spectroscopy study

**DOI:** 10.18632/aging.205585

**Published:** 2024-03-01

**Authors:** Xiaona Fu, Peng Sun, Xinli Zhang, Dongyong Zhu, Qian Qin, Jue Lu, Jing Wang

**Affiliations:** 1 Department of Radiology, Union Hospital, Tongji Medical College, Huazhong University of Science and Technology, Wuhan 430022, China; 2 Hubei Province Key Laboratory of Molecular Imaging, Wuhan 430030, China; 3 Clinical and Technical Support, Philips Healthcare, Beijing 100600, China

**Keywords:** anterior cingulate cortex, executive function, GABA, MEGA-PRESS, white matter hyperintensities

## Abstract

White matter hyperintensities (WMH) and gamma-aminobutyric acid (GABA) are associated with executive function. Multiple studies suggested cortical alterations mediate WMH-related cognitive decline. The aim of this study was to investigate the crucial role of cortical GABA in the WMH patients. In the 87 WMH patients (46 mild and 41 moderate to severe) examined in this study, GABA levels in the anterior cingulate cortex (ACC) and posterior cingulate cortex (PCC) assessed by the Meshcher-Garwood point resolved spectroscopy (MEGA-PRESS) sequence, WMH volume and executive function were compared between the two groups. Partial correlation and mediation analyses were carried out to examine the GABA levels in mediating the association between WMH volume and executive function. Patients with moderate to severe WMH had lower GABA+/Cr in the ACC (*p* = 0.034) and worse executive function (*p* = 0.004) than mild WMH patients. In all WMH cases, the GABA+/Cr levels in the ACC mediated the negative correlation between WMH and executive function (ab: effect = −0.020, BootSE = 0.010, 95% CI: −0.042 to −0.004). This finding suggested GABA+/Cr levels in the ACC might serve as a protective factor or potential target for preventing the occurrence and progression of executive function decline in WMH people.

## INTRODUCTION

White matter hyperintensities (WMH), referred to as white matter lesions or leukoaraiosis, are commonly defined as areas of high signal intensity on magnetic resonance imaging (MRI) T2-weighted or fluid-attenuated inversion recovery (FLAIR) images with low signal intensity on T1-weighted images [1]. WMH is one of the most important radiological manifestations of cerebral small vessel disease (CSVD), the main cause of cognitive impairment and vascular dementia [2, 3]. The incidence and severity of WMH are known to increase with age [4]. The prevalence of WMH ranges from 5% to 90%, depending on study design, study population, and rating scales [4]. In the general population, WMH affects about 90% of individuals aged 60 years or older [5], with more than 90% of those aged 80 years or older exhibiting some degree of WMH [4]. The pathological mechanism of WMH is complex, involving ischemia, demyelination, blood-brain barrier dysfunction, altered microglial expression, and inflammation [6].

The central nervous system (CNS) anatomically comprises gray matter and white matter based on tissue color. Gray matter mostly comprises neuronal cell bodies, glial cells, and dendrites, while white matter primarily comprises myelinated axonal fibers extending from the neuronal soma and glial cells [7]. White matter plays an important role in connecting various gray matter regions, enabling coordinated cognitive function within the brain. Executive function refers to multiple high-level cognitive processes required for goal-directed behavior [8]. Generally, higher executive function is associated with reduced risk of external or internal interference. Evidence suggests WMH are associated with cognitive impairment, particularly in terms of executive function [9–11]. As the volume of WMH increases, executive function tends to decline. Moreover, increasing evidence indicates that cortical thickness or gray matter volume mediates the declined memory and executive function detected in clinical WMH [12, 13].

Gamma-aminobutyric acid (GABA), an amino acid neurotransmitter in the CNS, plays a role in suppressing neuronal excitability and reducing energy consumption [14]. Multiple studies have shown that the homeostasis of the nervous system relies on the balance between excitation and inhibition [14, 15]. GABA levels are tightly associated with cognitive function [16, 17], and a genome-wide study demonstrated executive function is affected by GABAergic processes [18]. Proton Magnetic Resonance Spectroscopy (^1^H-MRS) is a non-invasive tool for detecting endogenous neurochemicals. It relies on the principle that radiofrequency signals emitted by hydrogen nuclear spins exhibit chemical specificity, manifesting as distinct peaks in the spectrum [19]. However, due to low brain GABA levels (approximately 1 mmol/L) and the overlap of GABA peaks with other metabolites’ peaks, detecting GABA levels accurately is hardly achieved by the traditional MRS [20]. The emerging Meshcher-Garwood point-resolved spectroscopy (MEGA-PRESS) sequence, incorporating the J-difference spectrum editing technology, is effective for rapid and accurate quantitation of GABA levels [21]. This tool also effectively captures glutamate-glutamine (Glx) signals due to structural and chemical similarities between Glx and GABA [22]. Current clinical studies examining CSVD have particularly focused on identifying appropriate imaging markers, including brain networks, WMH, cortical thickness, gray matter volume, and metabolism [23–25]. While studies have investigated neurochemical metabolites in WMH lesions and grey matter (18F)-fluorodeoxyglucose (FDG) in relation to WMH lesions [26, 27], the relationship between WMH and cortical GABA levels is rarely examined.

The anterior cingulate cortex (ACC) and posterior cingulate cortex (PCC) are critical brain regions of the limbic system. They were in the superior and posterior parts of the corpus callosum, respectively. Broadly speaking, the ACC is associated with emotion and executive function, and the PCC as an important node within the default mode network (DMN) connects with regions involved in memory, emotion and executive control [28–30]. Additionally, these two regions also exhibit metabolic differences. Research indicated that the PCC had greater acetylcholine receptor binding densities than the ACC [31]. Previous studies supported that cognitive function was sensitive to cerebral GABA concentrations in the frontal cortex. Moreover, GABA concentration in frontal and posterior regions continued to decline in later age, and decrease in GABA with age in the frontal region was more rapid in women than men [32, 33].

The purpose of the study was to investigate the crucial role of cortical GABA in the WMH patients from following three specific aspects: (i) to compare GABA levels in the ACC and the PCC and executive function between the mild WMH and moderate to severe WMH groups; (ii) to analyze correlations among WMH volume, GABA levels in the ACC and the PCC, and executive function; iii) to explore whether GABA levels in the ACC and the PCC mediate the association between WMH and executive function in WMH patients.

## MATERIALS AND METHODS

### Patients

**Figure 1:**
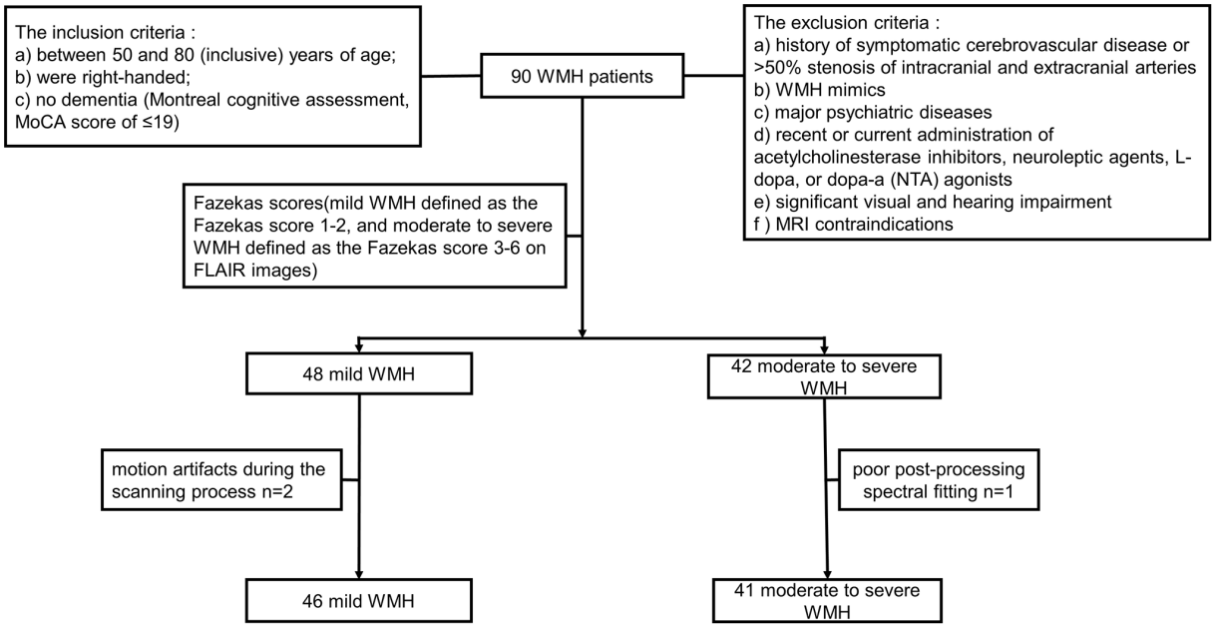
Flowchart for study recruitment.

The Ethics Committee of Union Hospital, Tongji Medical College, Huazhong University of Science and Technology approval and informed consent from each subject were obtained prior to study initiation. Ninety individuals, including 48 mild and 42 moderate to severe WMH cases were initially recruited from the neurology clinics of Union Hospital, Tongji Medical College, Huazhong University of Science and Technology. All participants underwent neuropsychological evaluation and MRI scanning. A radiology expert assessed all images based on the Fazekas score [34] (mild and moderate to severe WMH were defined as Fazekas scores of 1–2 and 3–6 on FLAIR images, respectively). Two mild WMH cases with motion artifacts during the scanning process and 1 moderate to severe WMH case with poor post-processing spectral fitting were excluded. Therefore, 46 mild and 41 moderate to severe WMH cases were finally included. All study participants (a) were between 50 and 80 (inclusive) years old; (b) were right-handed; (c) had no dementia (Montreal cognitive assessment, MoCA score ≤19 [35, 36]). Exclusion criteria were: (a) a history of symptomatic cerebrovascular disease or >50% stenosis of intracranial and extracranial arteries; (b) WMH mimics (e.g., neurodegenerative disorders, multiple sclerosis, trauma, infection, poisoning, hypoxic-ischemic encephalopathy, and leukodystrophy); (c) major psychiatric diseases (e.g., severe major depression, severe anxiety, and bipolar disorder); (d) recent or current administration of acetylcholinesterase inhibitors, neuroleptic agents, L-dopa, or dopa-a (nta) agonists; (e) significant visual or hearing impairment; (f) MRI contraindications, e.g., metal implants, pacemakers and claustrophobia. The study flowchart is depicted in Figure 1.

### Neuropsychological assessment

All individuals underwent neuropsychological assessment by a neuropsychologist. Global cognition was assessed by the Beijing version of the MoCA (MoCA-BJ) [37]. The auditory verbal learning test (AVLT) [38], including immediate recall, delayed recall, cued recall, and recognition, was utilized to examine individual episodic memory performance. Attention and executive function were assessed by the shape trail test (STT) [39], a modified version of the traditional trail-making test, and the Digit Span Test [40], which has two parts, i.e., forward digit span test (FDS) and backward digit span test (BDS). In which the FDS and STT-A measured attention whereas the BDS and STT-B measured execution [41]. However, some research used the BDS to assess working memory [42, 43], which is one of the types of executive function. Raw examination scores were z-transformed, with greater z-scores representing higher cognitive function. Subsequently, the z-score of every domain was obtained by averaging the z-scores of the related tests [44]. The emotional state was evaluated by the Hamilton Depression Rating Scale (HAMD) and the Hamilton Anxiety Rating Scale (HAMA). Individuals with HAMD score ≥24 [45] or HAMA score ≥29, indicating severe depression or anxiety, were excluded.

### Neuroimaging

All participants underwent scanning on a 3.0T scanner (Philips Achieva TX; Philips, Best, The Netherlands) utilizing a 32-channel phased-array head coil. The experimental protocol involved using magnetization-prepared turbo field echo sequences to acquire 3D high-resolution T1-weighted images with the following settings: repetition time (TR), 5.8 ms; echo time (TE), 2.7 ms; field of view (FOV), 220 × 220 × 180 mm^3^; voxel size, 1 × 1 × 1 mm^3^; acquisition time, 4:13 min; slice thickness, 1 mm. 3D FLAIR images were acquired utilizing a TSE sequence with the following settings: TR, 4800 ms; TE, 307 ms; FOV, 220 × 220 × 150 mm^3^; voxel size, 1 × 1 × 2 mm^3^; acquisition time, 1:41 min; slice thickness, 2 mm; flip angle, 90°; in-plane resolution, 1 × 1 mm^2^.

**Figure 2:**
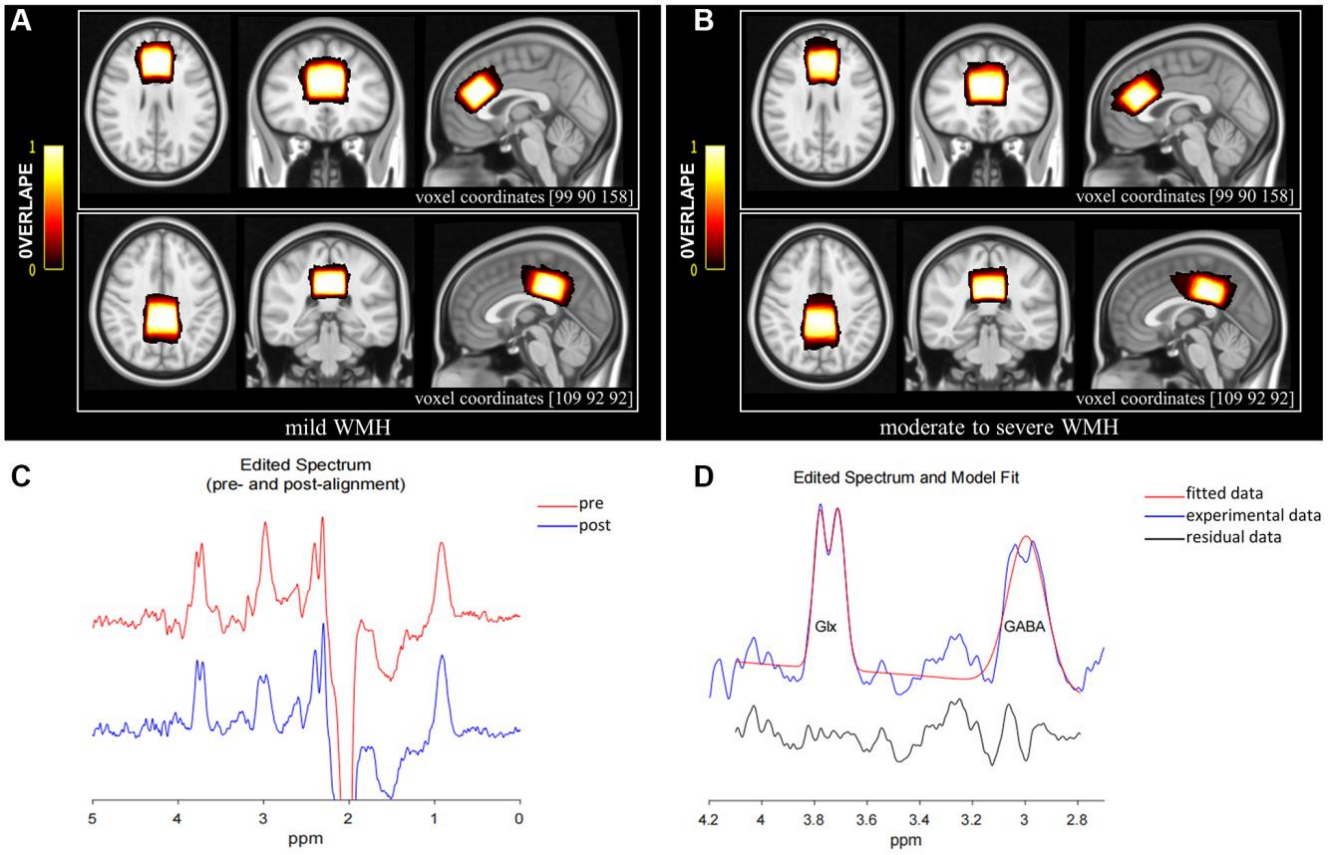
MRS voxel positions for mild WMH (**A**) and moderate to severe WMH participants (**B**). The bar color reflects the degree of overlap between individual voxels; the brighter the color, the higher the overlap. The coordinates for the transverse, coronal, and sagittal planes of the ACC are (90 90 158), while those for the PCC are (109 92 92). (**C**) GannetLoad output showing spectra pre (red line) and post (blue line) frequency and phase correction. (**D**) GannetFit output showing fitted GABA+/Cr and Glx/Cr signals: blue line, experimental data; red line, fitted data; black line, residual data. Abbreviations: ACC: anterior cingulate cortex; PCC: posterior cingulate cortex.

The MEGA-PRESS sequence, a J-difference spectrum editing technology, was employed to obtain GABA+ and Glx concentrations relative to creatine (Cr). It employed an interleaved acquisition protocol to obtain metabolite concentration, with frequency-selective and refocusing Gaussian pulses applied at 1.89 ppm (ON spectrum) and 7.46 ppm (OFF spectrum), respectively, during odd and even scanning. The ACC (volume of 30 × 30 × 20 mm³) and the PCC (volume of 30 × 30 × 20 mm³) were considered the regions of interest (ROIs) based on 3D high-resolution T1-weighted images (Figure 2A, 2B), avoiding lateral ventricles and the skull. The midsagittal plane was selected as the reference plane for voxel positioning. ACC and PCC voxels were in the superior and posterior parts of the corpus callosum, respectively, aligning with the shape of the corpus callosum and situated medial to the axial plane. The sequence settings were as follows: TR, 2000 ms; TE, 68 ms; 1024 points, averaging 160 (80 ON and 80 OFF resonance pulses); spectral bandwidth, 2000 Hz; acquisition time, 5:40 min per voxel (total scanning time of 11:20 min). Water suppression was carried out by the variable power and optimized relaxation delay (VAPOR) method.

### Data postprocessing

#### MRS processing

Head movement was suspected when artifacts, including line splitting and signals from outside the region of interest (e.g., subcutaneous lipid signals in brain MRS), were found in spectra [46]. Head motion artifacts were addressed by the following approaches: (1) head fixation with a sponge pad and noise-canceling headphones before the scan; (2) B0 shim correction during data acquisition; and (3) automatic frequency and phase correction, artifact suppression based on frequency deviation (>3 SD mean), and 3 Hz exponential line-broadening during data preprocessing.

The Gannet3.1 toolbox (https://github.com/richardedden/Gannet3.1), based on MATLAB, was utilized to quantitate metabolite signals (Figure 2C, 2D). Subtracting spectrum images (ON-OFF), the concentrations of metabolites were estimated by fitting Gaussian curves to the 3.02 ppm peak (GABA+) and double Gaussian curves to the 3.74 ppm (Glx) peak and scaling them relative to Cr, which is more stable and robust compared with water referencing [47, 48]. And these relative values may effectively reduce the systematic errors from inhomogeneities of both the B0 and B1 magnetic fields when using an external reference [49]. The signal detected by MEGA-PRESS at 3.02 ppm was referred to as “GABA+” instead of GABA because of the mixture of contributions from GABA, macromolecules, and homocarnosine. We maintained the full width at less than 20 Hz at half-maximum (FWHM) values to control the quality of MRS data. Meanwhile, individual metabolite ratios with FitError above 15% were excluded.

### Region of interest positioning and segmentation

Figure 2A, 2B illustrate average voxel positions for both voxels, after transformation to Montreal Neurological Institute (MNI152) space with the FSL software (FMRIB SOFTWARE LIBRARY, Oxford UK, FSL version 6.0). The 3D high-resolution T1-weighted images were transformed into the MNI space following the structural image processing pipeline from the UK Biobank [50]. We first reduced FOV of T1 images by cutting down non-brain tissue using BET [51] and FLIRT [52], in conjunction with the MNI152 “nonlinear 6th generation” standard-space T1 template15. This results in a reduced-FOV T1 head image and the linear transformation matrix from T1 space to MNI space. A non-linear registration to MNI152 space was carried out using FNIRT [53] to obtain the non-linear warp field. Then linear transformation matrix and non-linear warp field were combined to generate the final non-linear transformation, which allows the original T1 to be transformed into MNI152 space in a single step. The final non-linear transformation was then applied to the MEGA-PRESS ROIs in subject space from Gannet3.1 package to transform them to MNI152 space.

The proportions of white matter (WM), gray matter (GM), and cerebrospinal fluid (CSF) in the voxels affected metabolite levels in both groups. Therefore, 3D high-resolution T1-weighted images were first converted to the NIfTI format with MRIcroGL (https://www.nitrc.org/projects/mricrogl/), followed by the application of SPM12 (http://www.fil.ion.ucl.ac.uk/spm/software/spm12/) to segment and measure GM, WM, and CSF volumes within each MRS voxel. The GM/(GM + WM) ratio for each voxel was subsequently determined manually.

### WMH measurement

**Figure 3:**
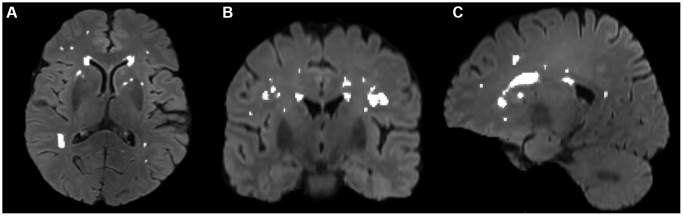
Segmentation of WMH volumes in the transverse (**A**), coronal (**B**), and sagittal (**C**) positions on FLAIR images. The white color represents the automatically segmented WMH range. WMH, white matter hyperintensities.

The volumes of white matter hyperintensities were derived with FSL’s Brain Intensity AbNormality Classification Algorithm (BIANCA) [54] following the UK Biobank image processing pipeline [50] and FMRIB’s Automated Segmentation Tool (FAST) [55]. The main procedures included: (a) generating a white matter mask with FAST, (b) generating a WMH mask with BIANCA in T1 space using a threshold of 0.8, which has achieved good segmentation performance according to visual check by an experienced neuro radiologist Dr. Wang, and (c) filtering non-WM voxels in the WMH mask with the white matter mask. Then, segmentation for every patient was manually checked and revised with ITK-SNAP (http://www.itksnap.org/) by Dr. Zhu. A representative segmentation of WMH volume is shown in Figure 3.

### Statistical analysis

#### Differential comparisons and correlation analysis

SPSS (version 25.0; SPSS Inc., Chicago, IL, USA) was used for all statistical analyses, and GraphPad Prism (version 9.4.0) was employed for graphing. Data distribution was assessed by the Shapiro-Wilk test. Differences between groups were assessed by the Mann-Whitney *U* test and independent samples *t* test. Count data were compared by the χ2 test. A general linear model was applied to determine group differences in metabolite ratios, and neurocognitive z-scores, with age, gender, and education in years as covariates. Partial correlation analyses were performed to determine the correlations of metabolites, WMH volume, and executive function, with age, gender, and education in years as covariates. *P* < 0.05 was considered statistically significant.

### Mediation analysis

Mediation analysis used PROCESS for SPSS v3.5 framework [56]. WMH volume was defined as the predictor (X), the metabolite ratios of the ACC and the PCC as the mediator (M), and cognitive function as the outcome (Y). This method partitions the total effect (denoted as c) shared by X and Y into two components: one mediated by M (indirect effect; ab) and the other independent of M (direct effect; c’). All associations of mediators with outcomes were determined after adjustment for age, gender, and education. We primarily examined the overall association between WMH volume and cognitive function (total effect; c). Secondly, WMH-metabolite ratios (path a) and metabolite ratios-cognitive function (path b) were independently assessed. We next tested the mediating effect of GABA on the association between WMH volume and cognitive function (path ab). Finally, the relationship between WMH volume and executive function was determined after removing the mediating effect (direct effect, c’). The bootstrap method (*n* = 5,000) was utilized for mediation analysis, with significant indirect effects defined as 95% confidence intervals (CI) fully above or below zero.

## RESULTS

### Demographic and neuropsychological parameters

The demographic data and neurocognitive properties of the participants are summarized in Table 1. The moderate to severe WMH group had elevated age compared with the mild WMH group (*p* = 0.013). No significant differences were detected in gender (*p* = 0.412), BMI (*p* = 0.858), education (*p* = 0.376), diabetes (*p* = 0.841), hypertension (*p* = 0.193), hyperlipemia (*p* = 0.879), smoking (*p* = 0.592) and drinking (*p* = 0.105) between the two groups. There were no differences in emotional state (HAMA, *p* = 0.507; HAMD, *p* = 0.197) and global cognition (MoCA, *p* = 0.834). The moderate to severe WMH group showed worse executive function (*p* = 0.004) than mild WMH groups; however, no differences were found in episodic memory (*p* = 0.141) and attention (*p* = 0.436), with age, gender, and education in years as covariates.

**Table 1:** Demographic, clinical and neuropsychological characteristics of participants.

	**mild WMH (*n* = 46)**	**moderate to severe WMH (*n* = 41)**	***p*-value**
Age (years)	62.07 ± 5.23	64.92 ± 5.31	0.013^*^
Gender (F/M)	34/12	27/14	0.412
BMI (kg/m^2^)	23.55 ± 2.60	23.66 ± 3.11	0.858
Edu (years)	10.5 (8–12)	11 (9–12)	0.376
Diabetes, *n* (%)	5 (10.9)	3 (7.3)	0.841
Hypertension, *n* (%)	14 (30.4)	18 (43.9)	0.193
Hyperlipemia, *n* (%)	15 (32.6)	14 (34.1)	0.879
Smoking, *n* (%)	8 (17.4)	9 (22.0)	0.592
Drinking, *n* (%)	6 (13.0)	11 (26.8)	0.105
WMH volume (cm^3^)	0.86 (0.49–1.26)	4.35 (2.70–7.99)	<0.001^***^
HAMA	3.5 (0.25–6.75)	2 (0–4.0)	0.507
HAMD	2.5 (0–5.75)	1 (0–4.0)	0.197
**Global cognition**			
MoCA	24(21.25–27)	25(22-27)	0.834
**Episodic memory**			
AVLT-immediate recall	14 (13–17)	14 (12–17.5)	0.151
AVLT-delayed recall	9.33 ± 4.50	8.41 ± 4.62	0.354
AVLT-cued recall	4.50 ± 2.44	3.85 ± 2.22	0.202
AVLT-recognition	21 (20–22)	19 (17–22)	0.287
**Attention**			
FDS	7 (6–8)	8 (6–8)	0.799
STT-A	62.65 ± 20.24	67.54 ± 21.18	0.275
**Executive function**			
BDS	4 (4–5)	4 (3–5)	0.054
STT-B	152.35 ± 35.76	174.66 ± 42.51	0.009^**^
**Neuropsychological z-scores^a^**			
Episodic memory	0.11 ± 0.81	−0.12 ± 0.88	0.141
Attention	0.07 ± 0.84	−0.08 ± 0.90	0.436
Executive function	0.22 ± 0.75	−0.25 ± 0.84	0.004^**^

Data are mean ± SD, number (percentage), or median (IQR, interquartile range). Abbreviations: WMH: white matter hyperintensities; HAMA: Hamilton anxiety scale; HAMD: Hamilton depression scale; MoCA: Montreal cognitive assessment; AVLT: Auditory Verbal Learning Test; BDS: Digit span test backward; FDS: Digit span test forward; STT: Shape Trail Test. ^a^Cognitive z-scores were corrected for age, sex and education level. ^*^p < 0.05, ^**^p < 0.01, ^***^p < 0.001.

### Metabolite concentrations

**Figure 4:**
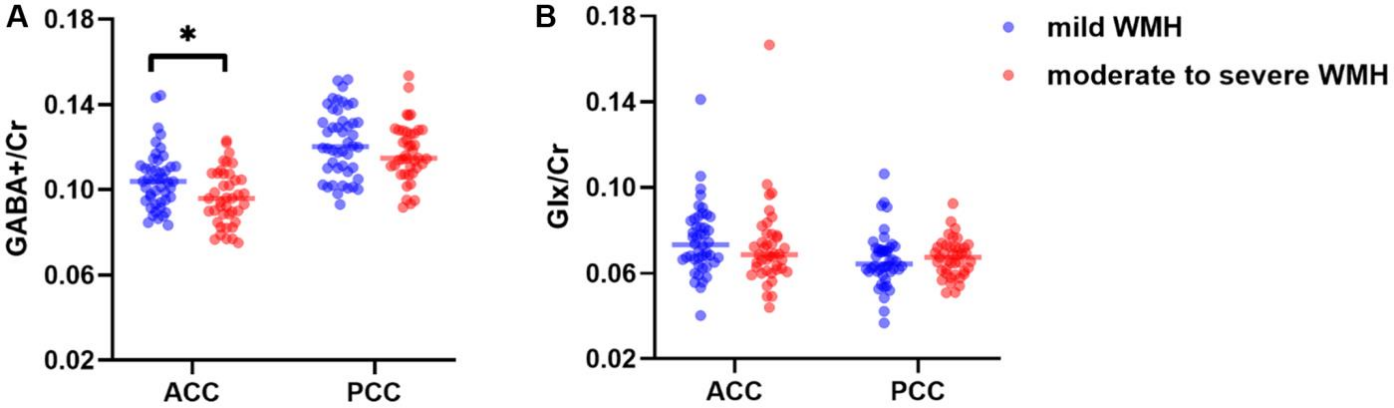
Distributions of GABA+/Cr (**A**) and Glx/Cr (**B**) levels in the ACC and the PCC. ^*^p < 0.05.

The metabolite data of all participants are shown in Table 2 and Figure 4 GABA+/Cr (*p* = 0.034) ratios were reduced in the moderate to severe WMH group compared with mild WMH cases, after adjustment for age, gender, and education. However, no significant differences were found in Glx/Cr levels in the ACC (*p* = 0.676) and in GABA+/Cr (*p* = 0.302) and Glx/Cr (*p* = 0.668) levels in the PCC.

**Table 2:** MRS analysis and tissue segmentation in the measured voxels.

	**mild WMH (*n* = 46)**	**moderate to severe WMH (*n* = 41)**	***p*-value**
**ACC**			
GABA+/Cr^a^	0.10 (0.09–0.11)	0.10(0.09–0.11)	0.034^*^
Glx/Cr^a^	0.07 (0.07–0.08)	0.07 (0.06–0.08)	0.676
GABA+ fitting errors (%)	8.00 (6.71–9.78)	8.17 (6.15–9.91)	0.683
Glx fitting errors (%)	7.62 (6.27–8.89)	7.11 (6.23–10.89)	0.766
FWHM (Hz)	9.16 (8.67–9.49)	9.16 (8.54–9.58)	0.990
GM/(GM + WM) (%)	54.02 ± 4.45	54.54 ± 5.54	0.637
**PCC**			
GABA+/Cr^a^	0.12 (0.11–0.13)	0.12 (0.11–0.13)	0.302
Glx/Cr^a^	0.06 (0.06–0.07)	0.07 (0.06–0.07)	0.668
GABA+ fitting errors (%)	6.66 (5.65–7.60)	7.00 (5.97–7.78)	0.575
Glx fitting errors (%)	7.08 (6.08–9.18)	7.13 (6.00–7.99)	0.560
FWHM (Hz)	8.97 (8.57–9.25)	8.91 (8.79–9.16)	0.710
GM/(GM + WM) (%)	59.28 ± 3.36	58.15 ± 4.91	0.218

Data are mean ± SD, or median (IQR, interquartile range). Abbreviations: WMH: white matter hyperintensities; GABA+: GABA plus co-edited macromolecules and homocarnosine; Glx: glutamate-glutamine; FWHM: full-width at half-maximum; GM: gray matter; WM: white matter. ^a^After correction for age, gender, and education level. Data are median (IQR, interquartile range). ^*^p < 0.05, ^**^p < 0.01, ^***^p < 0.001.

### ROI segmental data and MRS quality

In the moderate to severe WMH group, GM/(GM + WM) averaged 54.54% and 58.15% in the ACC and the PCC, respectively, versus 54.02% and 59.28% in mild WMH cases, respectively (Table 2). Meanwhile, no significant differences were found in GM/(GM + WM), fitting errors of metabolite ratios, and FWHM between the two groups in each voxel (all *p* > 0.05) (Table 2).

### Relationships among WMH burden, neurometabolites, and cognitive function

**Figure 5:**
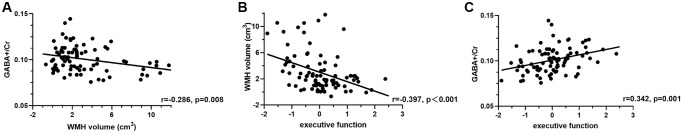
Partial correlations among WMH volume (**A**, **B**), GABA+/Cr levels in the ACC (**A**, **C**), and executive function (**B**, **C**) in all WMH participants, with age, gender, and education as covariates.

In all WMH patients, a negative correlation was found between WMH volume and GABA levels in the ACC (r = −0.286, *p* = 0.008, Figure 5A), but not in the PCC (*p* > 0.05). Glx levels in both regions were not associated with WMH burden in the whole brain (all *p* > 0.05). WMH burden was negatively correlated with executive function (r = −0.397, *p* < 0.001, Figure 5B), but not with attention (r = −0.177, *p* = 0.108) and memory (r = −0.005, *p* = 0.967).

In all WMH patients, significant positive correlations were found between GABA+/Cr in both regions and executive function (ACC, r = 0.342, *p* = 0.001; PCC, r = 0.241, *p* = 0.027; Figure 5C and Supplementary Table 1) and attention (ACC, r = 0.237, *p* = 0.030; PCC, r = 0,225, *p* = 0.040; Supplementary Table 1), after adjustment for age, gender, and education. However, GABA+/Cr levels in both regions were not correlated with memory. In addition, no associations were found between Glx/Cr in both regions and cognitive function (all *p* > 0.05), except for Glx/Cr that was correlated with memory in the PCC (r = 0.295, *p* = 0.007, Supplementary Table 1).

### Mediation analysis of WMH volume on cognitive performance through neurometabolites

**Figure 6:**
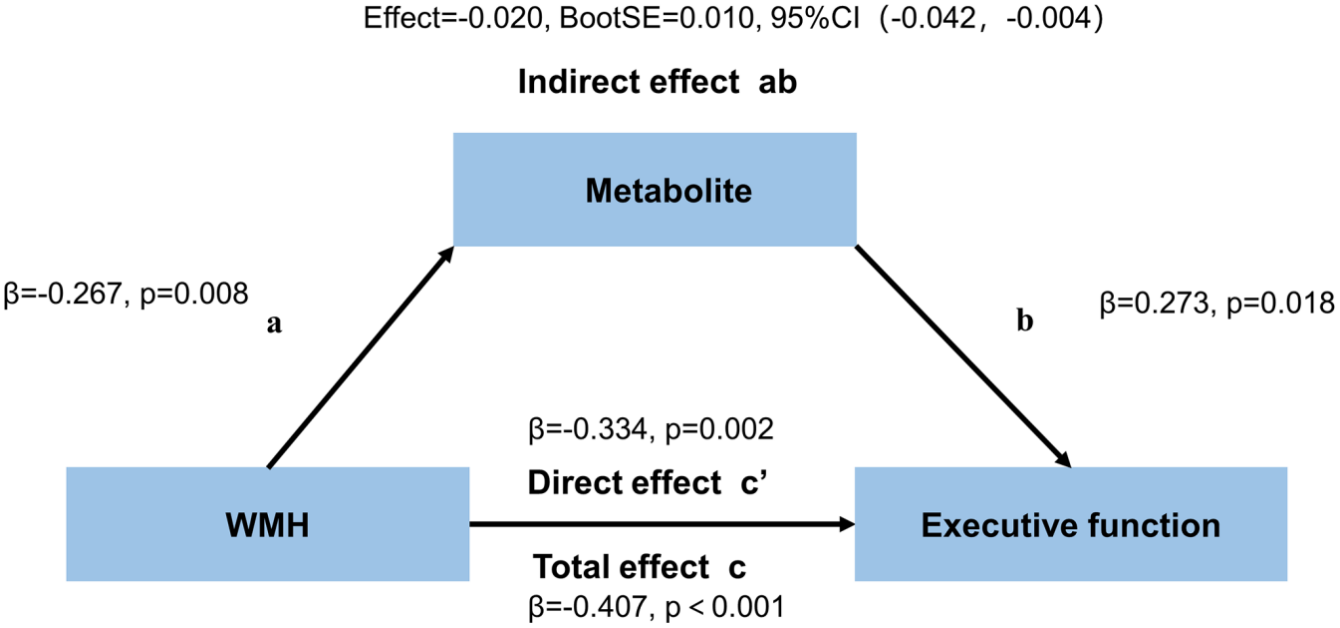
Mediation effects of GABA+/Cr levels in the ACC on the association between WMH volume and executive function.

The neurometabolites mediating the association of WMH with executive function in the ACC are summarized in Figure 6 and Supplementary Table 2. The total and direct effects of WHH volume on executive function mediated through GABA+/Cr in the ACC were significant (c: effect= −0.109, β= −0.407, *p* < 0.001, c’: effect= −0.090, β= −0.334, *p* = 0.002) in all WMH patients. The indirect effect of this model was also significant (ab: effect= −0.020, BootSE = 0.010, 95% CI: −0.042 to −0.004). However, GABA+/Cr levels in the PCC and Glx/Cr amounts in both regions had no mediation effects. There were no mediating effects of WMH volume on attention and memory through GABA+/Cr or Glx/Cr in both regions (Supplementary Tables 3 and 4).

## DISCUSSION

This study demonstrated the moderate to severe WMH group had reduced executive function compared with mild WMH cases. GABA+/Cr levels in the ACC were significantly decreased in the moderate to severe WMH group compared with mild WMH patients. However, no significant differences were found in Glx/Cr in the ACC and in both metabolites in the PCC. In addition, WMH volume was correlated with GABA levels in the ACC, as well as executive function in all WMH participants. This study revealed GABA levels in the ACC mediated the association between WMH and executive function in WMH patients, while GABA levels in the PCC and Glx in both regions had no mediating effects.

This study demonstrated the moderate to severe WMH patients were older and had worse executive function compared with mild WMH group, corroborating previous studies [4, 13]. Research indicated that executive function referred to a series of high-level cognitive control abilities necessary for goal achievement, such as working memory, response inhibition, selection among competing options and task switching [19]. And some studies used BDS to assess working memory [42, 43]. Therefore, we need incorporate a broader range of scales to comprehensively evaluate executive function in the future study. However, there were no significant differences in episodic memory and attention between the two groups, contradicting previous research [57, 58]. This discrepancy may be attributed to the small sample size and the use of different assessment scales. In addition, WMH volume was negatively correlated with executive function in WMH patients. White matter comprises structural connections linking various regions of gray matter throughout the brain. These white matter bundles are capable of transmitting information that is essential for diverse cognitive functions, including memory, attention, and execution [7]. A report demonstrated WMH impair fiber connections within the brain, reducing executive function [24]. Previous studies showed a negative correlation between WMH burden and executive function [11, 30], with increased WMH burden and declined executive function. These findings jointly suggested a significant correlation between WMH and executive function, which was further supported by the current findings. This correlation may be attributed to the fact that elevated WMH volume results in more pronounced damage to the pathways responsible for information transmission.

There was a significance between group difference in GABA+/Cr levels in the ACC. Homeostasis of the central nervous system depends on the balance between excitatory and inhibitory neurotransmitters [14, 15]. Previous reports demonstrated reduced GABA or Glx levels in the ACC and the PCC in individuals with multiple disorders, including multiple sclerosis, schizophrenia, and mild cognitive impairment [59–61]. This study found that moderate to severe WMH patients had severely impaired executive function. Therefore, the decreased GABA levels in the ACC obtained in this study were possibly due to CNS disturbance caused by moderate to severe WMH. In physiological mechanisms, GABAergic/glutamatergic neurons depend on a continuous supply of glutamine (Gln, a precursor of glutamate and GABA) from astrocytes, for further synthesis of GABA or Glu. System A transporters in neurons and system N transporters in astrocytes mediate Gln transport between astrocytes and neurons [14]. The decreased GABA+/Cr in this study was probably due to neuronal loss, reduced GABA synthesis, or altered astrocyte cycle of GABA, Glu, and Gln. We found no significant differences in metabolites in the PCC, which could be attributed to the small sample size or population heterogeneity. Therefore, large studies are required to further investigate differences in neurotransmitters between the two groups.

We also revealed a significant inverse relationship between GABA levels in the ACC and WMH volume, as well as a positive correlation between GABA levels and executive function in all WMH patients. Imaging studies reported disrupted brain networks and brain atrophy mediate the association of WMH with cognitive impairment [13, 62, 63]. Additionally, the relationship between WMH and cortical FDG uptake was examined in elderly hypertensive patients with subjective memory symptoms [64], demonstrating a negative correlation between Fazekas score and cortical FDG uptake. These findings jointly emphasized the tight connection between gray and white matter in the brain, which was further supported by the current results using the imaging marker GABA. However, the relationship between execution and GABA has been controversial in previous reports. A study showed higher GABA levels in dorsal ACC are associated with improved executive function [29]. The current results were highly consistent with this finding. However, a previous report found Glx/total Cr (tCr) levels in the dorsolateral prefrontal cortex, but not GABA/tCr, are significantly associated with executive function in individuals with amnestic mild cognitive impairment (aMCI) [65]. Another study detected no significant associations of GABA levels in the ACC and the PCC with executive function determined by the symbol digit modalities test in aMCI patients [61]. The discrepancy was likely due to differences in scales used for executive function assessment as well as the locations of ROIs. Additionally, the WMH patients involved in this study included non-demented individuals, which may also lead to differences in results compared to MCI studies.

We demonstrated that GABA levels in the ACC mediated the relationship between WMH and executive function, complementary to many previous studies that revealed disrupted brain networks and brain atrophy mediated the relationship between WMH and cognitive impairment [13, 23]. The neurovascular unit is important in CSVD, and comprises neurons, glial cells, and cerebral blood vessels jointly working to maintain microenvironmental homeostasis and ensure normal brain function [66, 67]. White matter primarily consists of myelinated axonal fibers, and the pathological mechanism of WMH is complex [6]. Therefore, the current results suggest that CSVD may induce the disruption of white matter fiber bundles, which appeared as WMH on FLAIR images. Due to secondary degeneration, the damaged fiber bundles also affected the axonal cytoskeleton [68] and the cortex, where the cortex was characterized by high amounts of neuronal cell bodies, including GABA neurons and astrocytes. Subsequently, alterations in GABA levels drive changes in executive function [18]. This finding not only suggested the ACC as an important region in executive function but also indicated that GABA content in the ACC may serve as a protective factor or a potential therapeutic target for impaired executive function in WMH populations.

This study had several limitations despite notable advancements in the current findings. First, this was a cross-sectional study with a small sample size. Future large and longitudinal studies are required to further validate the reliability of these results and to explore the dynamic changes of metabolite levels, WMH volume, and execution function in WMH patients. Secondly, expanding the scope of future studies by incorporating additional brain regions, such as orbitofrontal cortex, dorsolateral prefrontal cortex, is crucial to provide a more comprehensive understanding of intricate relationships among WMH burden, GABA, and executive function. Additionally, the measured metabolites did not exclusively reflect pure GABA and Glu, as GABA+ was a combination of GABA, macromolecules, and homocarnosine, while Glx was a mixture of Glu and Gln. To precisely detect “pure GABA” in WMH patients by GABA-edited MRS, it may be necessary to use novel macromolecular inhibition techniques since it is unlikely that changes in homocarnosine are the primary factor determining the observed alterations. Moreover, multiple studies have subdivided WMH into different cerebral subregions, and future investigation should also assess the relationships among WMH, GABA, and executive function in specific subregions of the brain.

## CONCLUSION

We demonstrated that individuals with moderate to severe WMH have reduced executive function and decreased GABA+/Cr levels in the ACC compared with mild WMH group. Moreover, we revealed that GABA in the ACC mediates the association between WMH and executive function in WMH patients. These findings not only indicated that GABA+/Cr levels in the ACC have a vital role, but also suggested GABA levels in the ACC may serve as a protective factor or potential target for preventing the occurrence and progression of executive function impairment in WMH patients.

## Supplementary Materials

Supplementary Tables
