# Supplementary material for: GABA in the anterior cingulate cortex mediates the association of white matter hyperintensities with executive function: a magnetic resonance spectroscopy study
Source: Aging (Albany NY). 2024 Mar 1;16(5):4282–98. doi: 10.18632/aging.205585 (PMC10968699; doi:10.18632/aging.205585)
Supplement: Supplementary Tables [file aging-16-205585-s001.pdf]

SUPPLEMENTARY TABLES

Supplementary Table 1. Partial correlations between metabolites, WMH volume, and cognitive function.

	Executive function		Attention		Episodic memory	
	r	*p*	r	*p*	r	*p*
ACC(GABA+/Cr)	0.342	**0.001**	0.237	**0.030**	0.121	0.274
ACC(Glx/Cr)	−0.035	0.749	−0.112	0.310	−0.052	0.639
PCC(GABA+/Cr)	0.241	**0.027**	0.225	**0.040**	0.184	0.094
PCC(Glx/Cr)	0.029	0.790	0.034	0.761	0.295	**0.007**
WMH volume (cm^3)	−0.397	**<0.001**	−0.177	0.108	−0.005	0.967

Abbreviations: GABA+: GABA plus co-edited macromolecules and homocarnosine; Glx: glutamate-glutamine; ACC: anterior cortical cortex; PCC: posterior cortical cortex.

Supplementary Table 2. Mediation effects of metabolites on the association between WMH volume and executive function.

Metabolites	Total effect (c)			Direct effect (c')			a		b		Indirect effect (ab)		
	Effect	β	*p*	Effect	β	*p*	β	*p*	β	*p*	Effect	BootSE	95% CI
ACC(GABA +/Cr)	−0.109	−0.407	**<0.001**	−0.090	−0.334	**0.002**	−0.267	**0.008**	0.273	**0.018**	−0.020	0.010	**(−0.042, −0.004)**
ACC(Glx/Cr)				−0.111	−0.416	**<0.001**	−0.111	0.325	−0.080	0.439	0.002	0.004	(−0.003, 0.012)
PCC(GABA+ /Cr)				−0103	−0.386	**<0.001**	−0.104	0.359	0.202	**0.045**	−0.006	0.007	(−0.022, 0.008)
PCC(Glx/Cr)				−0.110	−0.409	**<0.001**	0.041	0.714	0.047	0.656	0.001	0.003	(−0.004, 0.009)

Abbreviations: GABA+: GABA plus co-edited macromolecules and homocarnosine; Glx: glutamate-glutamine; ACC: anterior cortical cortex; PCC: posterior cortical cortex.

Supplementary Table 3. Mediation effects of metabolites on the association between WMH volume and attention.

Metabolites	Total effect (c)			Direct effect (c')			a		b		Indirect effect (ab)		
	Effect	β	*p*	Effect	β	*p*	β	*p*	β	*p*	Effect	BootSE	95% CI
ACC(GABA+/Cr)	−0.049	−0.171	0.108	−0.033	−0.115	0.291	−0.267	**0.008**	0.210	0.074	−0.016	0.011	(−0.041, 0.002)
ACC(Glx/Cr)				−0.052	−0.185	0.083	−0.111	0.325	−0.126	0.226	0.004	0.005	(−0.002, 0.017)
PCC(GABA+/Cr)				−0.043	−0.151	0.151	−0.104	0.359	0.197	0.055	−0.006	0.008	(−0.024, 0.007)
PCC(Glx/Cr)				−0.049	−0.173	0.106	0.041	0.714	0.039	0.709	0.001	0.004	(−0.006, 0.012)

Abbreviations: GABA+: GABA plus co-edited macromolecules and homocarnosine; Glx: glutamate-glutamine; ACC: anterior cortical cortex; PCC: posterior cortical cortex.

Supplementary Table 4. Mediation effects of metabolites on the association between WMH volume and episodic memory.

Metabolites	Total effect (c)			Direct effect (c')			a		b		Indirect effect (ab)		
	Effect	β	p	Effect	β	p	β	p	β	p	Effect	BootSE	95% CI
ACC(GABA+/Cr)	−0.001	−0.005	0.967	0.009	0.033	0.777	−0.267	**0.008**	0.140	0.261	−0.010	0.010	(−0.033, 0.008)
ACC(Glx/Cr)				−0.003	−0.010	0.927	−0.111	0.325	−0.052	0.636	0.002	0.004	(−0.006, 0.011)
PCC(GABA+/Cr)				0.004	0.014	0.897	−0.104	0.359	0.181	0.095	−0.005	0.008	(−0.026, 0.006)
PCC(Glx/Cr)				−0.005	−0.017	0.877	0.041	0.714	0.294	0.007	0.003	0.009	(−0.015, 0.022)

Abbreviations: GABA+: GABA plus co-edited macromolecules and homocarnosine; Glx: glutamate-glutamine; ACC: anterior cortical cortex; PCC: posterior cortical cortex.
